# Stabilized Molybdenum Trioxide Nanowires as Novel Ultrahigh‐Capacity Cathode for Rechargeable Zinc Ion Battery

**DOI:** 10.1002/advs.201900151

**Published:** 2019-05-14

**Authors:** Xinjun He, Haozhe Zhang, Xingyu Zhao, Peng Zhang, Minghua Chen, Zhikun Zheng, Zhiji Han, Tingshun Zhu, Yexiang Tong, Xihong Lu

**Affiliations:** ^1^ Key Laboratory of Engineering Dielectric and Applications (Ministry of Education) Harbin University of Science and Technology Harbin 150080 China; ^2^ MOE of the Key Laboratory of Bioinorganic and Synthetic Chemistry The Key Lab of Low‐Carbon Chem & Energy Conservation of Guangdong Province School of Chemistry Sun Yat‐Sen University Guangzhou 510275 P. R. China; ^3^ School of Environment and Civil Engineering Guangdong Engineering and Technology Research Center for Advanced Nanomaterials Dongguan University of Technology Dongguan 523808 China

**Keywords:** cathodes, durability, high‐energy, MoO_3_, Zn ion batteries

## Abstract

Exploration of high‐performance cathode materials for rechargeable aqueous Zn ion batteries (ZIBs) is highly desirable. The potential of molybdenum trioxide (MoO_3_) in other electrochemical energy storage devices has been revealed but held understudied in ZIBs. Herein, a demonstration of orthorhombic MoO_3_ as an ultrahigh‐capacity cathode material in ZIBs is presented. The energy storage mechanism of the MoO_3_ nanowires based on Zn^2+^ intercalation/deintercalation and its electrochemical instability mechanism are particularly investigated and elucidated. The severe capacity decay of the MoO_3_ nanowires during charging/discharging cycles arises from the dissolution and the structural collapse of MoO_3_ in aqueous electrolyte. To this end, an effective strategy to stabilize MoO_3_ nanowires by using a quasi‐solid‐state poly(vinyl alcohol)(PVA)/ZnCl_2_ gel electrolyte to replace the aqueous electrolyte is developed. The capacity retention of the assembled ZIBs after 400 charge/discharge cycles at 6.0 A g^−1^ is significantly boosted, from 27.1% (in aqueous electrolyte) to 70.4% (in gel electrolyte). More remarkably, the stabilized quasi‐solid‐state ZIBs achieve an attracting areal capacity of 2.65 mAh cm^−2^ and a gravimetric capacity of 241.3 mAh g^−1^ at 0.4 A g^−1^, outperforming most of recently reported ZIBs.

## 1 Introduction

With the fast‐growing demand in energy‐consuming products like electric vehicles and portable electronics, it is imperative to explore advanced energy storage technologies with high energy, good safety, and affordable cost.^1^, ^2^, ^3^, ^4^, ^5^, ^6^, ^7^, ^8^ As one of the most promising energy storage devices, aqueous zinc‐ion batteries (ZIBs) has gained ever‐increasing attention on account of its outstanding safety, high theoretical capacity (Zn: ≈820 mAh g^−1^), low cost as well as environmental benignity.^9^, ^10^, ^11^, ^12^, ^13^, ^14^, ^15^ In addition, the aqueous ambient of the electrodes also accelerates the ion diffusion owing to the high ionic conductivity, which enables them a better rate capability.^16^, ^17^, ^18^ Despite these advanced features, one of the biggest block for ZIBs developed to date is their relatively low capacity in terms of the substantially inferior capacity of cathode materials than Zn anode.^19^, ^20^, ^21^ To this end, numerous research efforts have been focused on the exploiting of high‐capacity cathode materials over the past few decades.^21^, ^22^, ^23^, ^24^ A great variety of materials including MnO_2_,^25^, ^26^, ^27^, ^28^ ZnMn_2_O_4_,^17^ Zn*_x_*Mo_6_S_6_,^29^ ZnHCF,^30^ Zn_0.25_V_2_O_5_·nH_2_O,^31^ VS_2_,^20^ NiOOH,^32^ and Co_3_O_4_ 9 have been reported as promising cathodes with good electrochemical performance. For example, Wu and co‐workers employed ultrathin porous Co_3_O_4_ nanosheets which was electrodeposited on Ni foam as a cathode material, and it delivered a capacity of 0.32 mAh cm^−2^ in Zn//Co_3_O_4_ battery (0.05 A g^−1^).^9^ Pan and co‐workers used a hydrothermal method to synthesize α‐MnO_2_ nanofibers, which exhibited a capacity of 1.43 mAh cm^−2^ at 0.1 A g^−1^.^33^ Nevertheless, the capacity of reported ZIBs is still unsatisfactory for future high‐energy electronics. Developing new cathode materials with high capacity and excellent durability are yet challenging and highly desirable.

Orthorhombic MoO_3_ nanomaterials serve as versatile electrodes for Li ion battery and supercapacitors because of their multiple valence states and unique layered structures.^13^, ^34^, ^35^, ^36^, ^37^ In previous studies, MoO_3_ has shown its reversible Li‐ion insertion/extraction ability.^36^, ^37^ Given that the similar ion radius of Zn^2+^ (0.75 Å) and Li^+^ (0.76 Å), it is expected that the MoO_3_ holds great promise as high‐performance electrode for ZIBs.^9^, ^26^ However, to our knowledge, there are few reports on the exploration of molybdenum oxides as electrode in ZIBs. In this work, we first investigated the electrochemical properties of orthorhombic MoO_3_ nanowires as Zn^2+^ storage electrode and demonstrated their implementation as high‐capacity cathode in ZIBs. The energy storage behavior of the orthorhombic MoO_3_ based on Zn^2+^ intercalation/deintercalation mechanism as well as its unstable mechanism were systematically unraveled by comprehensive characterizations. More importantly, an effective approach of using a quasisolid state electrolyte to stabilize MoO_3_ nanowires was developed. Electrochemical studies revealed that the ZIBs based on our MoO_3_ nanowire cathode in a quasisolid state poly(vinyl alcohol)(PVA)/ZnCl_2_ gel electrolyte achieve an ultrahigh capacity of 2.65 mAh cm^−2^ (243.1 mAh g^−1^) at 0.4 A g^−1^ and an impressive energy density of 14.4 mWh cm^−3^. Additionally, the cycling stability of this quasisolid state ZIB device is significantly improved compared to the device with aqueous electrolyte, maintaining more than 70.4% of its initial capacity after 400 cycles.

## 2 Results and Discussion

**Figure 1:**
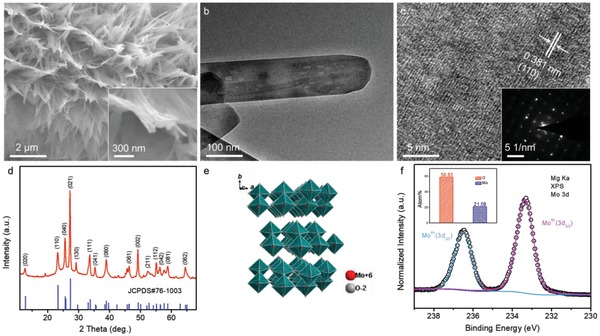
a) SEM images, b) TEM image, c) HRTEM image, d) XRD pattern, e) layered crystal structure, and f) core‐level Mo 3d of XPS spectrum of the MoO_3_ nanowires. The insets in (c,f) are its corresponding SAED pattern and the atom% of Mo and O, respectively.

MoO_3_ nanowires were synthesized through a quick and facile seed‐assisted hydrothermal method (details in Experimental Section). To characterize the morphology of the as‐synthesized nanowire, scanning electron microscopy (SEM) and transmission electron microscopy (TEM) were carried out. As shown in **Figure** **1**a, MoO_3_ nanowires with a diameter of about 100 nm are vertically grown on fibers of carbon cloth, and these nanowires intertwine to form bundles. Figure 1b displays the typical TEM image of the MoO_3_ nanowires, presenting its wire‐like morphology. The diameter of the nanowire is around 100 nm, which is well in agreement with SEM observation. Lattice fringes are observed in the high resolution TEM (HRTEM) image (Figure 1c), showing the polycrystalline nature of these MoO_3_ nanowires. This is further validated by the bright diffraction spots of its corresponding selected area electron diffraction (SAED) pattern (Figure 1c, inset). The lattice fringe with a *d*‐spacing of about 0.38 nm is attributed to (110) plane of orthorhombic MoO_3_ (JCPDS#76‐1003).

To explore the crystal structure and valance state of Mo, we conducted powder X‐ray diffraction (XRD) and X‐ray photoelectron spectroscopy (XPS) measurements. The representative XRD pattern of the MoO_3_ sample is collected in Figure 1d. All of the diffraction peaks can be well assigned to orthorhombic MoO_3_ (JCPDS#76‐1003) with space group of Pbnm(62), indicating the as‐obtained product is highly pure. The corresponding crystalline structure is shown in Figure 1e. [MoO_6_] octahedra share edges in *c*‐direction and are linked by corners in *a*‐direction to construct a layer in *ac*‐plane.^36^, ^37^ Then, these parallel layers stack through van der Waals force along *b*‐direction to form MoO_3_ layer architecture. It is believed that such unique layered structure is highly favorable for ion intercalation/deintercalation. Figure S1 of the Supporting Information presents the XPS survey spectrum of the MoO_3_ sample. Only three elements (Mo, O, and C) are detected on the surface of MoO_3_ sample, further indicating the high purity of these nanowires. The core‐level Mo 3d XPS spectrum can be deconvoluted into two peaks, as shown in Figure 1f. The peaks at the binding energy of 233.36 and 236.51 eV are the characteristic Mo 3d_5/2_ and 3d_3/2_ peaks of Mo^6+^.^38^ In addition, the ratio of Mo and O atom% obtained from XPS result is about 1:3 (inset in Figure 1f). All these findings adequately support that the as‐synthesized product is polycrystalline orthorhombic MoO_3_ nanowires.

**Figure 2:**
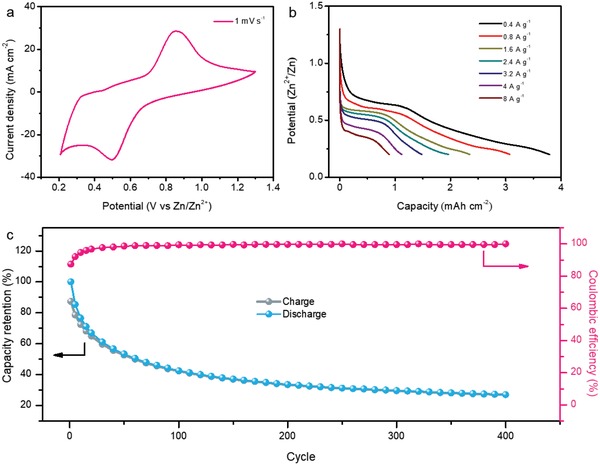
a) CV curves of the aqueous Zn//MoO_3_ battery in 2M ZnCl_2_ electrolyte at 1 mV s^−1^. b) Galvanostatic charge and discharge curves of Zn//MoO_3_ battery at different current densities. c) Lifespan of the aqueous device at current density of 6.0 A g^−1^.

An aqueous ZIB device was assembled to evaluate the electrochemical properties of the as‐obtained MoO_3_ nanowires, with a commercial zinc plate as anode and aqueous 2 M ZnCl_2_ solution as electrolyte (named as Zn//MoO_3_ battery). **Figure** **2**a presents the typical cyclic voltammetry (CV) curve of this MoO_3_ nanowires at a scan rate of 1 mV s^−1^. A couple of well‐defined redox peaks at around 0.85 and 0.50 V versus Zn^2+^/Zn is clearly identified, which shows an evident battery behavior. The obvious plateaus in galvanostatic charge–discharge curves collected at different current densities also identify the redox reactions (Figure 2b). Additionally, the calculated areal capacity of this Zn//MoO_3_ battery reaches 3.79 mAh cm^−2^ (344.8 mAh g^−1^ based on the mass of the MoO_3_), substantially larger than most of the developed ZIBs.^9^, ^17^, ^20^, ^33^ However, this Zn//MoO_3_ battery is found to suffer from severe capacity loss. As given by Figure 2c, its capacity declines rapidly in the first several cycles and only 27.1% of the initial capacity is preserved after 400 charge–discharge cycles, which makes it to be far away from practical application.

**Figure 3:**
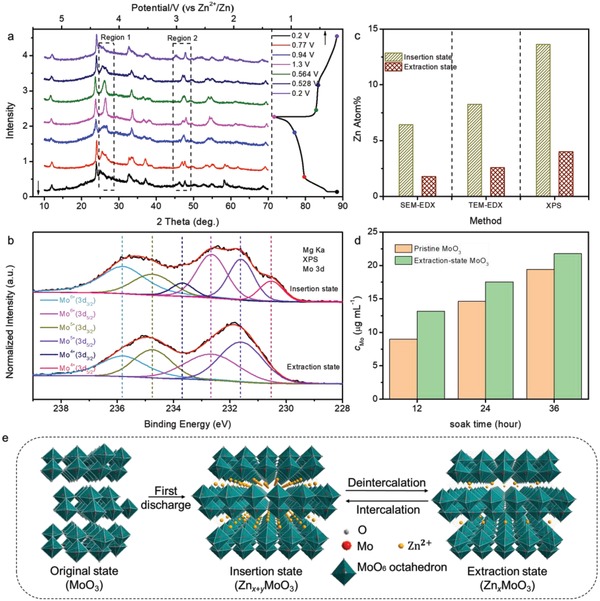
a) XRD pattern of MoO_3_ at different voltage. b) Core‐level Mo 3d XPS spectra and c) Zn Atom% obtained by different methods of MoO_3_ nanowires in insertion (0.2 V) and extraction (1.3 V) state. d) *C*_Mo_ for aqueous electrolyte after pristine MoO_3_ and extraction‐state MoO_3_ were immersed for different time. e) Schematic illustration of the Zn^2+^ intercalation/deintercalation mechanism for orthorhombic MoO_3_ electrode.

To elucidate the mechanism and the origin of the instability of the Zn//MoO_3_ battery, the componential and structural evolution of the orthorhombic MoO_3_ cathode during the charge–discharge process were meticulously investigated by the ex‐situ XRD, XPS, TEM‐energy dispersive X‐ray spectroscopy (TEM‐EDX) and UV–Visible spectroscopy analyses. XRD test was conducted under different voltages in the first and second charge–discharge cycles. In the first discharging segment, the set of (0*k*0) reflections including (020), (040), and (060), obviously shift toward lower positions when the voltage decreases from 0.64 to 0.50 V (Figure S2, Supporting Information). This fact suggests the expansion of the interlayer spacing due to the Zn^2+^ intercalation. As the voltage further decreases to 0.2 V, the continuous expansion of (020) interplanar spacing is observed whereas (040) and (060) reflections vanish gradually. According to the Bragg equation, the interlayer distance of (020) is calculated to increase from 6.9 to 7.3 Å during the whole discharging process. Moreover, the disappearance of (040) and (021) peaks indicates that Zn^2+^ not only inserts into the empty sites of the inter layer (ac plane) but also exists between the adjacent MoO_6_ octahedrons.^39^, ^40^ In the second charge/discharge cycle, all of the peaks in XRD pattern show invertible shift during charge/discharge process (**Figure** **3**a). For example, the peak at 46.2° in region 2 shifts to 47.1° when the voltage increases from 0.2 to 1.3 V, which suggests the shrink of the MoO_3_ lattice. This can be ascribed to different Zn^2+^/vacancy ordering patterns when the Zn^2+^ inserts into and extracts from the cathode.^31^ Additionally, after this charging process, the XRD pattern has not recovered from discharged state to original state, which indicates that a part of inserted Zn^2+^ remained in the MoO_3_ lattice. More XRD patterns of extraction‐state MoO_3_ during cycling have been collected in Figure S3 (Supporting Information). It can be seen that the pattern of first cycle has changed a lot compared with initial MoO_3_ and gradually become stabilized in subsequent cycles, suggesting the formation of solid electrolyte interface (SEI) layer at the initial cycles.^36^, ^37^ Simultaneously, Figure S4 of the Supporting Information shows that the zinc content of the extraction‐state MoO_3_ keeps increasing in the first four cycles, which confirms the above conclusion and it also explains the slightly lower Columbic efficiency for the first several charge/discharge cycles.

Figure 3b compares the core levels Mo 3d XPS spectra of the MoO_3_ nanowires under insertion (0.2 V) and extraction (1.3 V) states. At 0.2 V, the exclusive existence of Mo^4+^ (3*d*_3/2_: 235.82 eV), Mo^5+^ (3*d*_3/2_: 234.77 eV), and Mo^6+^ (3*d*_3/2_: 233.67 eV) evidently shows its deep discharging nature.^38^, ^41^ When the voltage is applied to 1.3 V, the Mo^4+^ peaks fades away, indicating the Mo^4+^ gradually turns to its higher valence state (Mo^6+^ and Mo^5+^) with Zn^2+^ constantly extracts out from the framework. The existence of Mo^5+^ in the extraction state indicates that Zn^2+^ has not totally deintercalated from MoO_3_ framework. Core‐level Zn 3*p* XPS spectrum of electrode in pristine, insertion, and extraction state were given in Figure S5 (Supporting Information). Upon discharging, a substantial leap of Zn 2*p* (2*p*_3/2_: 1022.2 eV) signal is recorded, which confirms the Zn^2+^ intercalation and deintercalation mechanism. Similarly, Figure 3c shows the Zn atom% of insertion state (0.2 V) and extraction state (1.3 V) obtained by three different methods, again revealing the intercalation and deintercalation of Zn^2+^ ions. All of these methods presented that Zn atom% of the insertion state in MoO_3_ nanowires is more than twice as much as that of the extraction state. Combined with TEM‐Mapping images (Figure S6, Supporting Information) where Zn^2+^ is homogenously distributed in the insertion/extraction sample, it is deduced that Zn^2+^ uniformly intercalates into the whole MoO_3_ cathode upon discharging. Meanwhile, the existence of zinc signal in extraction‐state MoO_3_ also suggests that Zn^2+^ has not completely extracted from the cathode, which is well consistent with XRD and other characterizations. Based on the above results, the overall reactions of our Zn//MoO_3_ battery can be summarized as

(1) $$\mathrm{Anode:}\;\mathrm{Zn}↔{\mathrm{Zn}}^{\mathrm{2+}}+{\mathrm{2e}}^{-}$$

(2) $$\begin{array}{c} \mathrm{Cathode:}\;{\mathrm{MoO}}_{3}+\left(x+y\right){\mathrm{Zn}}^{\mathrm{2+}}+2\left(x+y\right)e^{-}\to {\mathrm{Zn}}_{x+y}{\mathrm{MoO}}_{3}\\ \;\;\;\;\;\;\left(\mathrm{The}\;\mathrm{first}\;\mathrm{discharge}\;\mathrm{cycle}\right) \end{array}$$

(3) $$\begin{array}{l} {\mathrm{Zn}}_{x+y}{\mathrm{MoO}}_{3}↔{\mathrm{Zn}}_{x}{\mathrm{MoO}}_{3}+y{\mathrm{Zn}}^{2+}+2ye^{-}\\ \;\;\;\;\;\;\left(\mathrm{The}\;\mathrm{following}\;\mathrm{charge/discharge}\;\mathrm{cycles}\right) \end{array}$$

SEM and UV–Visible spectroscopy were carried out to probe into the poor cycling stability of MoO_3_ cathode in aqueous electrolyte. SEM and element mapping (Figure S7, Supporting Information) was operated on MoO_3_ under insertion and extraction states. In extraction state, the wire morphology with a diameter of around 100 nm is well retained. However, after Zn^2+^ insertion, a sheet‐like structure with a thickness of 100 nm is captured. Combining with the corresponding element mapping data, we observed that the extraction state MoO_3_ has much lower Zn element ratio than insertion state, which is also consistent with the XRD and TEM mapping results. To further uncover the morphology change phenomenon, we have conducted ex‐situ SEM to test samples in different voltages (corresponding to different Zn^2+^ intercalation ratio) in one charge–discharge cycles. As shown in Figure S8 of the Supporting Information, when the electrode charged to 0.77 V where starts the charging plateau, a sheet morphology is presented. With the deintercalation of Zn^2+^, those sheets gradually separate and finally change back to nanowires (at 0.94 V and finally to 1.3 V). On the contrary, with the processing of intercalation of Zn^2+^, the nanowires slowly aggregate to form bundles and finally become nanosheets in the insertion‐state MoO_3_. Moreover, the sheet morphology also occurs during charge and discharge between 4 and 0.4 A g^−1^ current density (Figure S9, Supporting Information), and it is more obvious at low current density, which may because more Zn^2+^ insert into MoO_3_ at a low current density and thus leads to a bigger morphology change. Indeed, the detailed morphology transformation mechanism is still unclear, and relative work is ongoing.

The concentration of Mo species in electrolyte (*C*_Mo_) was also recorded by UV–Visible spectroscopy to investigate the dissolution situation of the active material. The standard curve of Mo concentration was provided in Figure S10 (Supporting Information). Figure 3d compares the *C*_Mo_ of aqueous electrolyte after pristine and extraction‐state MoO_3_ cathodes were soaked for different periods of time (Spectra given in Figure S11 of the Supporting Information). It shows that the corresponding *C*_Mo_ of extraction‐state MoO_3_ is obviously higher than pristine MoO_3_. For example, the *C*_Mo_ values for pristine and extraction‐state MoO_3_ after 36 h are 19.39 and 21.76 µg mL^−1^, respectively. These results imply that MoO_3_ will be spontaneously dissolved in water even without applying a voltage, and the electrode with Zn^2+^ existence becomes more vulnerable to water. In this regard, one way to improve the cycling performance of the device is to suppress the severe dissolution of the cathode.

**Figure 4:**
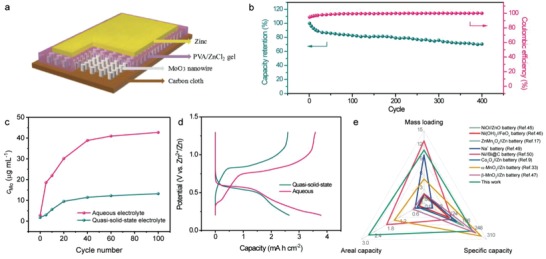
a) Schematic illustration of the quasisolid state Zn//MoO_3_ battery. b) Cycling performance of the quasisolid state Zn//MoO_3_ battery at a current density of 6.0 A g^−1^. c) Calculated *C*_Mo_ for aqueous and quasisolid state electrolyte after different cycles. d) Charge/discharge curves of the aqueous and quasisolid state Zn//MoO_3_ batteries at current density of 0.4 A g^−1^. e) The capacities of the quasisolid state Zn//MoO_3_ battery and some recently reported works.^9^, ^17^, ^33^, ^45^, ^46^, ^47^, ^49^, ^50^

Based on the above analyses, the aqueous electrolyte was replaced by a quasisolid state polyvinyl alcohol (PVA)/ZnCl_2_ based electrolyte to improve the stability of the MoO_3_ cathode. To demonstrate our hypothesis, a quasisolid state Zn//MoO_3_ battery was fabricated, and the schematic diagram of this battery is illustrated in **Figure** **4**a. The electrochemical durability of this quasisolid state Zn//MoO_3_ battery was probed by galvanostatic charge–discharge measurement at 6.0 A g^−1^ (Figure 4b). As expected, benefitting from the substantially lower water content and slower ion diffusion, this quasisolid state Zn//MoO_3_ battery yields remarkably enhanced stability compared to the aqueous Zn//MoO_3_ battery. Specifically, it is able to keep more than 70.4% of its original capacity after 400 cycles, dramatically higher than the aqueous one (27.1%) and comparable to the recently reported Zn ion batteries. Meanwhile, compared to aqueous device, the Coulombic efficiency of the Zn//MoO_3_ battery at the initial few cycles is also greatly improved in quasisolid state electrolyte (Figures 2c and 4b), suggesting its electrochemical reaction is more reversible. In addition, the *C*_Mo_ of aqueous and quasisolid state electrolytes after different charge/discharge cycles were also monitored when the MoO_3_ cathodes were applied to fabricate devices. Figure S12 of the Supporting Information presents the UV–Visible spectra of two kinds of electrolytes after different charge/discharge cycles, while the corresponding calculated the *C*_Mo_ values are shown in Figure 4c. For both electrolyte, the Mo concentration keeps increasing with the proceeding of charging and discharging. After 100 cycles, the detected *C*_Mo_ of aqueous electrolyte is 42.73 µg mL^−1^, which indicates that the dynamic charge/discharge behavior vastly accelerates the dissolution of MoO_3_. More importantly, the *C*_Mo_ values of the quasisolid state electrolyte are significantly lower than those of the aqueous electrolyte. For instance, after 100 cycles, the *C*_Mo_ in quasisolid state electrolyte is only 13.22 µg mL^−1^, much smaller than 42.73 µg mL^−1^ of the aqueous electrolyte. The significantly decreased Mo concentration verifying the important role of quasisolid state electrolyte in alleviating the dissolution of MoO_3_ and improving the cycling stability of MoO_3_ cathode.

Figure 4d compares the charge–discharge curves of the aqueous and quasisolid state Zn//MoO_3_ battery at a current density of 0.4 A g^−1^. A couple of charge and discharge plateaus at around 0.82/0.59 V are clearly captured for both devices with aqueous or quasisolid state electrolyte, which suggests that their energy storage mechanisms are similar. This conclusion is also supported by Figure S13 of the Supporting Information, a similar sheet morphology is also characterized after Zn^2+^ insertion in quasisolid state electrolyte. Moreover, the Zn atom content in MoO_3_ electrode using quasisolid state electrolyte had been measured by XPS in Figure S14 (Supporting Information). It is found that the zinc atom content shows an invertible change from 5.1% to 2.0% and finally to 6.4% at 0.2 V, 1.3 V, and back to 0.2 V, respectively. This dynamic change also confirms its Zn^2+^ intercalation–deintercalation mechanism. Compared to aqueous device, the capacity of quasisolid state device shows a visible drop, which can be ascribed to the slower ion diffusion of the quasisolid state battery. To confirm it, the Nyquist plots of the aqueous and quasisolid state Zn//MoO_3_ batteries are collected in Figure S15 (Supporting Information). Both of them contain two parts: the high‐frequency semicircular loop and the low‐frequency straight line, which are relative to charge transfer and ion diffusion, respectively. They are well fitted with the equivalent circuit that inserted in Figure S15 of the Supporting Information, where *R*_ct_ means the charge transfer resistance, *Z*_w_ represents the Warburg impedance while CPE is the constant phase element and *R*_e_ is the total ohmic resistance.^9^, ^42^, ^43^ In comparison with the aqueous Zn//MoO_3_ battery, the quasisolid state one exhibits much lower steep slope in the Warburg region, clearly showing that the ion diffusion rate in quasisolid state electrolyte become much slower. Additionally, the *R*_ct_ value of the battery in quasisolid state electrolyte is 61.2 Ω, which is just slightly larger than 57.9 Ω for aqueous electrolyte. It suggests that the electrolyte substitution has negligent effect on the electron transportation rate for the electrode. Despite a visible drop of capacity, this quasisolid state battery still delivers impressive capacities of 2.65 mAh cm^−2^ and 241.3 mAh g^−1^, which are considerably surpassing most of the recently developed ZIBs and other aqueous batteries (Figure 4e),^9^, ^17^, ^33^, ^44^, ^45^, ^46^, ^47^, ^48^, ^49^, ^50^ such as Ni(OH)_2_//FeO*_x_* battery (0.227 mAh cm^−2^ and 126 mAh g^−1^ at 1.5 A g^−1^),^46^ NiO//ZnO battery (0.26 mAh cm^−2^ and 197.9 mAh g^−1^ at 0.38 A g^−1^),^45^ Zn//Co_3_O_4_ battery (0.324 mAh cm^−2^ and 162 mAh g^−1^ at 1 A g^−1^),^9^ Zn//ZnMn_2_O_4_ battery (0.3 mAh cm^−2^ and 150 mAh g^−1^ at 0.05 A g^−1^),^17^ Zn//β‐MnO_2_ battery (0.516 mAh cm^−2^ and 258 mAh g^−1^ at 0.2 A g^−1^),^47^ Zn//ɑ‐MnO_2_ battery (1.425 mAh cm^−2^ and 285 mAh g^−1^ at 0.1 A g^−1^),^33^ Na^+^ battery (0.43 mAh cm^−2^ and 43 mAh g^−1^ at 0.1 A g^−1^),^49^ and Ni//Bi@C battery (1.79 mAh cm^−2^ and 139 mAh g^−1^ at 0.31 A g^−1^).^50^ Particularly, the mass loading of the MoO_3_ nanowires is up to 11.1 mg cm^−2^. Such high specific capacity achieved by this MoO_3_ electrode also reflects its prominent electrochemical performance. In addition, a high capacity retention of 38.1% (1.01 mAh cm^−2^) is exhibited when the current density is increased to 4 A g^−1^, suggesting its excellent performance (Figure S16, Supporting Information). Moreover, the rate performance shows quite similar behavior after being replaced by an aqueous electrolyte with quasisolid state electrolyte (Figure S16, Supporting Information).

**Figure 5:**
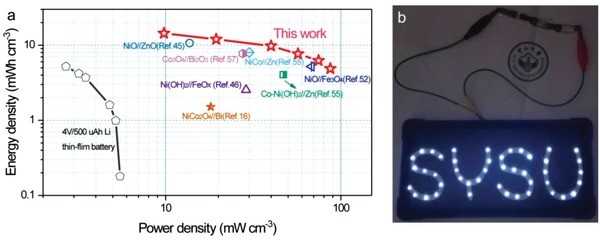
a) Ragone plots of quasisolid state Zn//MoO_3_ battery with the comparison of other reported electrochemical storage devices.^16^, ^45^, ^46^, ^51^, ^52^, ^55^, ^57^ b) Picture displays the LED lamps powered by quasisolid state Zn//MoO_3_ batteries.

Ragone plots in **Figure** **5**a compares the volumetric energy densities and power densities of our quasisolid state Zn//MoO_3_ battery with other published aqueous batteries and asymmetric supercapacitors (ASCs). Profiting by the high capacity, the as‐fabricated quasisolid state Zn//MoO_3_ battery at a power density of 9.79 mW cm^−3^ can afford a maximum energy density of 14.4 mWh cm^−3^, which greatly exceeds the value of previously developed aqueous batteries,^16^, ^44^, ^45^, ^46^, ^51^, ^52^, ^53^, ^54^, ^55^, ^56^ including Ni(OH)_2_//FeO*_x_* (2.54 mWh cm^−3^),^46^ NiCo_2_O_4_//Bi battery (1.51 mWh cm^−3^),^16^ Zn//Co‐Ni(OH)_2_ (4.05 mWh cm^−3^),^51^ NiO//Fe_3_O_4_ (5.24 mWh cm^−3^),^52^ Co_3_O_4_//Bi_2_O_3_ battery (7.74 mWh cm^−3^),^57^ Zn//NiCo (8 mWh cm^−3^),^55^ and NiO//ZnO battery (10.67 mWh cm^−3^).^45^ Furthermore, a relatively large energy density of 4.9 mWh cm^−3^ is still delivered by our device even at a high power density of 87.2 mW cm^−3^, disclosing its good rate performance. Additionally, as shown in Figure 5b, our quasisolid state Zn//MoO_3_ batteries connected in series can power 45 LEDs (3.0 V), showing its great practical value.

## 3 Conclusion

In summary, our studies disclose that the dominant reasons for the electrochemical instability of MoO_3_ nanowires are the severe destruction and dissolution of active material. We further demonstrated that the employment of a PVA/ZnCl_2_ quasisolid state electrolyte could effectively fix these issues. ZIBs based on the MoO_3_ nanowire cathode with quasisolid state electrolyte delivers a decent capacity retention of more than 70.4% after 400 cycles, substantially superior to the one with aqueous electrolyte (27.1%). Moreover, the capacity and energy density of this quasisolid state battery achieve up to 2.65 mAh cm^−2^ (243.1 mAh g^−1^) at 0.4 A g^−1^ and 14.4 mWh cm^−3^ at 9.79 mW cm^−3^, respectively, outperforming most of other ZIBs. This work presents the first example of quasisolid state electrolyte stabilized MoO_3_ as an ultrahigh capacity cathode material for ZIBs, which will give new insights in the exploration of advanced energy storage systems.

## 4 Experimental Section

*Preparation of MoO_3_*: MoO_3_ nanowires were synthesized on carbon cloth through a seed‐assisted hydrothermal approach. First, Na_2_MoO_4_ · 2H_2_O (2.5 g) was dissolved in the solution containing HCl (5 mL, 37 wt. %) and deionized (DI) water (20 mL). Then, the clean carbon cloth (70 mg) were immersed into the aforesaid solution for 5 min and blow‐dried with the help of air blower. Immediately, the dry carbon cloth was further heated in the oven (340 °C) for another 5 min to form MoO_3_ nanoparticles on it. The precursor solution was prepared by dissolving (NH_4_)_6_Mo_7_O_24_ (0.5 g) in the mixture of concentrated HNO_3_ (3 mL, 65 wt. %) and DI water (17 mL). Next, the prepared carbon cloth was immersed into the precursor solution followed by being transferred to a Teflon‐lined stainless steel autoclave (25 mL). The autoclave was heated in a drying oven with a heating speed of 10 °C min^−1^ to 140 °C for 5 min and then cooled down to room temperature. Afterward, the attained carbon cloth were thoroughly washed with DI water and dried, which formed the sample of MoO_3_ nanowires. The mass loading of the MoO_3_ nanowires deposited on carbon cloth is around 11.1 mg cm^−2^ (BT25S, 0.01 mg).

*Fabrication of Quasi Solid State* Zn//MoO_3_ *Battery*: MoO_3_@carbon cloth and zinc plate was assembled together with a separator (NKK separator, Nippon Kodoshi Corporation) between them to form our battery. This PVA/ZnCl_2_ gel was made by mixing 2 g polyvinyl alcohol, 10 mL deionized water, and 10 mL 2m ZnCl_2_ (5 µL concentrated HCl was added to make ZnCl_2_ fully dissolved), and heated at 85 °C for 1 h under vigorous stirring. The electrodes and separator were soaked in this gel before assembling. After that, they were piled up layer‐by‐layer followed by a solidification process to remove excessive water. Finally, the battery was packaged with an area of 0.5 cm^2^ and thickness of 0.08 cm.

*UV–Visible Spectroscopy (Dissolution Test)*: Standard curve. First, Na_2_MoO_4_ · 2H_2_O was dissolved into 1.84 mol L^−1^ H_2_SO_4_ to prepare solutions with a Mo concentration of 0, 5, 10, 20, 30, 50 µg mL^−1^. Simultaneously, 100 mL 250 g L^−1^ KSCN, 100 mL 50 g L^−1^ ascorbic acid, 100 mL 1.84 mol L^−1^ H_2_SO_4_, and 5 mL 0.4 g L^−1^ CuSO_4_ were mixed together and gently shook to form chromogenic agent. Then, 6 mL of chromogenic agent was added to 5 mL of different concentration of Mo solutions. Wait for 10 min and then measure absorbance at the wavelength of 470 nm. The value was averaged after three times of parallel experiments. The standard curve was obtained by drawing and fitting Absorbance–*C*_Mo_ curve. Electrolyte sample preparation. 5 mL mixed concentrated H_2_SO_4_ and HNO_3_ (1:4 for volume ratio) was added to filtered electrolytes. Then, the solutions were heated at 200 °C until fully dried. 2 mL mixed concentrated H_2_SO_4_ and HNO_3_ were added into the dried solution and was heated again. The residue was dissolved by 1.84 mol L^−1^ H_2_SO_4_ to make a final volume of 50 mL. The next procedures can be referred to the standard Mo solution test.

*Material Characterization and Electrochemical Measurement*: The morphology, microstructures, and compositions of the electrode materials were analyzed using field‐emission SEM (FE‐SEM, JSM‐6330F), TEM (FEI Tecnai G^2^ F30), XPS (ESCALab250, Thermo VG), and XRD (D8 ADVANCE). CV, galvanostatic charge/discharge measurements, GITT, and electrochemical impedance spectroscopy were conducted employing an electrochemical workstation (CHI 760D). The electrochemical performances of Zn//MoO_3_ Batteries were tested in a two‐electrode system in a 2m ZnCl_2_ electrolyte. The dissolution test was conducted on a UV–vis spectrometer (SHIMADZU UV‐2600 220 V CH).

## Conflict of Interest

The authors declare no conflict of interest.

## Supporting information

Supplementary
