# Supplementary material for: Stabilized Molybdenum Trioxide Nanowires as Novel Ultrahigh‐Capacity Cathode for Rechargeable Zinc Ion Battery
Source: Adv Sci (Weinh). 2019 May 14;6(14):1900151. doi: 10.1002/advs.201900151 (PMC6662057; doi:10.1002/advs.201900151)
Supplement: Supplementary file 1 — Supplementary [file ADVS-6-1900151-s001.pdf]

Copyright WILEY-VCH Verlag GmbH & Co. KGaA, 69469 Weinheim, Germany, 2019.

ADVANCED SCIENCE
Open Access

Supporting Information

for *Adv. Sci.,* DOI: 10.1002/advs.201900151

Stabilized Molybdenum Trioxide Nanowires as Novel Ultrahigh-Capacity Cathode for Rechargeable Zinc Ion Battery

Xinjun He, Haozhe Zhang, Xingyu Zhao, Peng Zhang, Minghua Chen, Zhikun Zheng, Zhiji Han, Tingshun Zhu, Yexiang Tong, and Xihong Lu**

Copyright WILEY-VCH Verlag GmbH & Co. KGaA, 69469 Weinheim, Germany, 2019.

Supporting Information

Stabilized Molybdenum Trioxide Nanowires as Novel Ultrahigh-Capacity Cathode for Rechargeable Zinc ion Battery

Xinjun He,[a,b] *Haozhe Zhang,*[b] *Xingyu Zhao,*[a] *Peng Zhang,*[c] *Minghua Chen,**[a] *Zhikun Zheng,*[b] *Zhiji Han,*[b] *Tingshun Zhu,*[b] *Yexiang Tong,*[b] *and Xihong Lu**[a,b]

[a]*Key Laboratory of Engineering Dielectric and Applications (Ministry of Education), Harbin University of Science and Technology, Harbin 150080, China. E-mail: mhchen@hrbust.edu.cn (M. Chen)*

[b]*MOE of the Key Laboratory of Bioinorganic and Synthetic Chemistry, The Key Lab of Low-carbon Chem & Energy Conservation of Guangdong Province, School of Chemistry, Sun Yat-Sen University, Guangzhou 510275, P. R. China.*

E-mail: luxh6@mail.sysu.edu.cn (X. Lu)

[c]*School of Environment and Civil Engineering, Guangdong Engineering and Technology Research Center for Advanced Nanomaterials, Dongguan University of Technology, Dongguan 523808, China.*

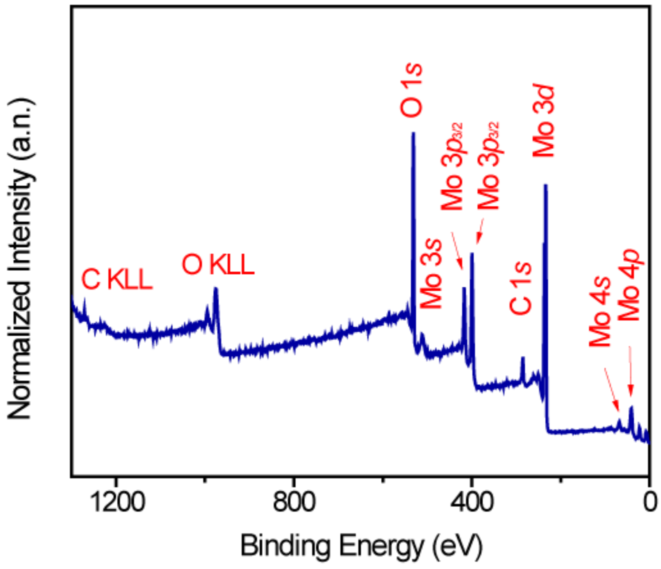

Figure S1. XPS survey spectrum of MoO_3 nanowire sample.

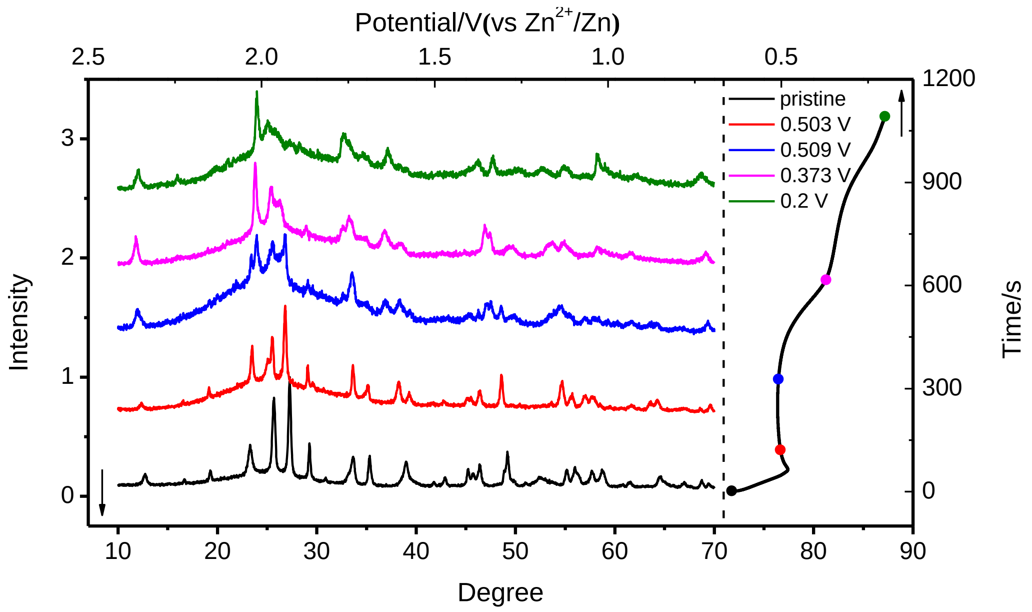

Figure S2. XRD pattern of sample at different voltages in the first discharge segment.

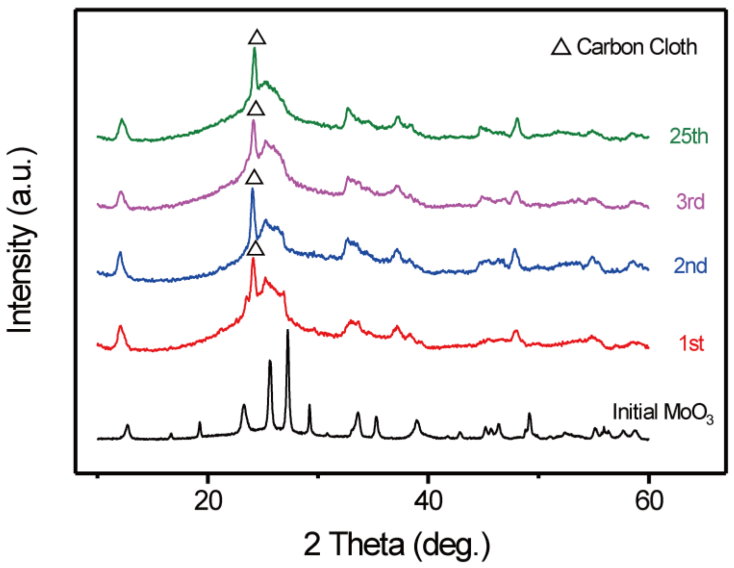

Figure S3. XRD pattern of MoO_3 at original state or after different charging and discharging cycles.

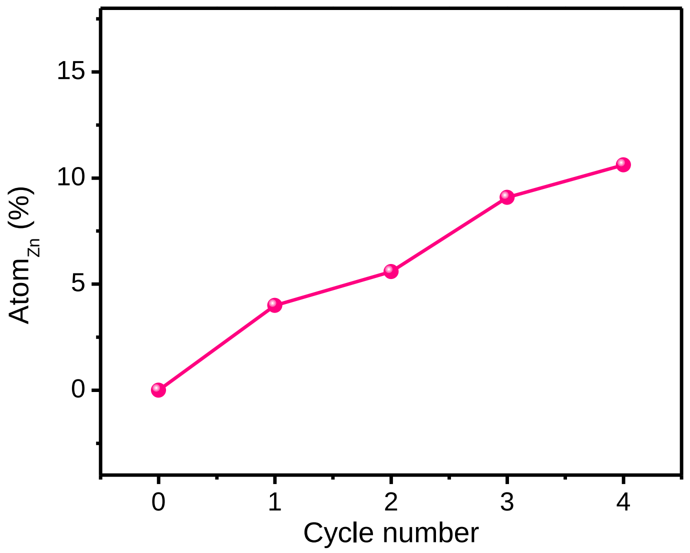

Figure S4. Zinc content in extraction-state MoO_3 after different charge-discharge cycles.

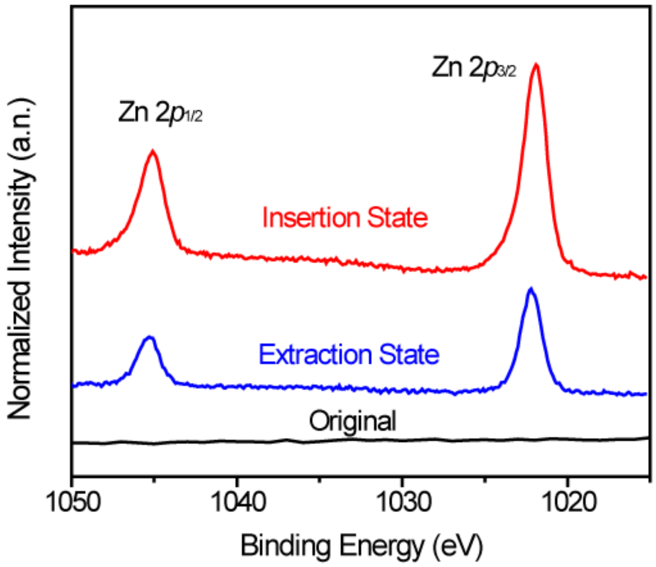

Figure S5. Zn 2p XPS spectra for MoO_3 at pristine, extraction and insertion state.

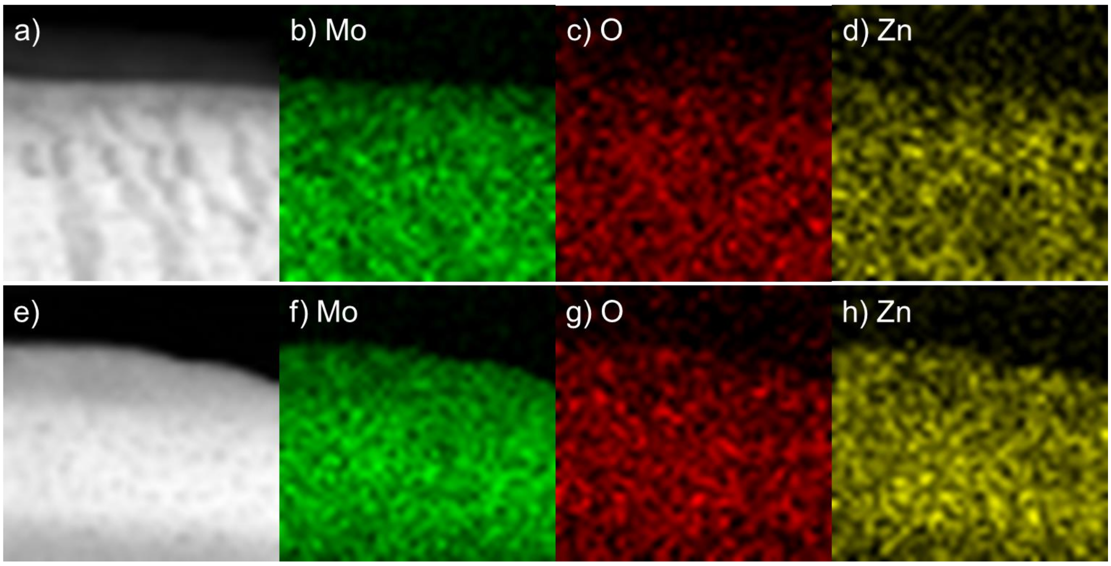

Figure S6. TEM-Mapping of (a-d) extraction state and (e-h) insertion state of MoO_3 electrode.

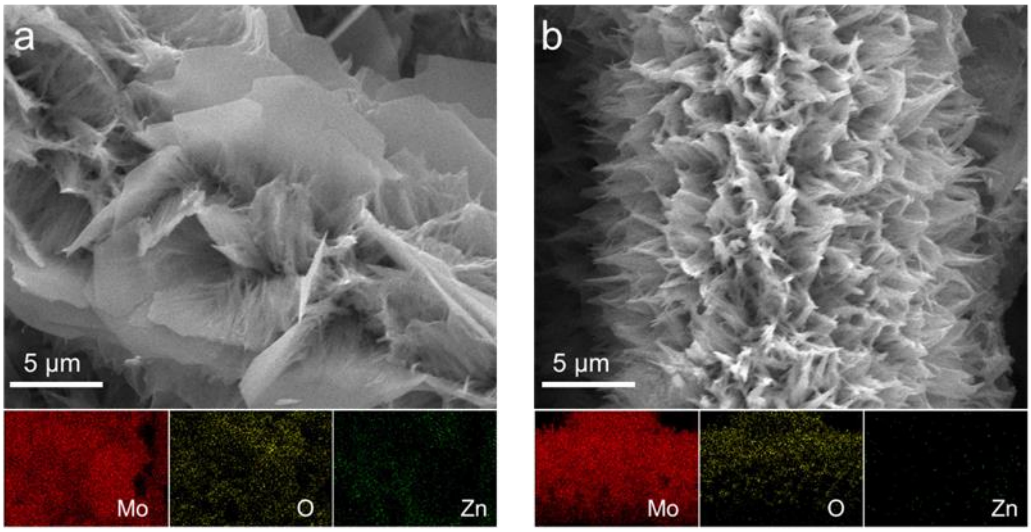

Figure S7. SEM images of MoO_3 at (a) 0.2 V (insertion state) and (b) 1.3 V (extraction state). Nether figures are corresponding SEM mapping data.

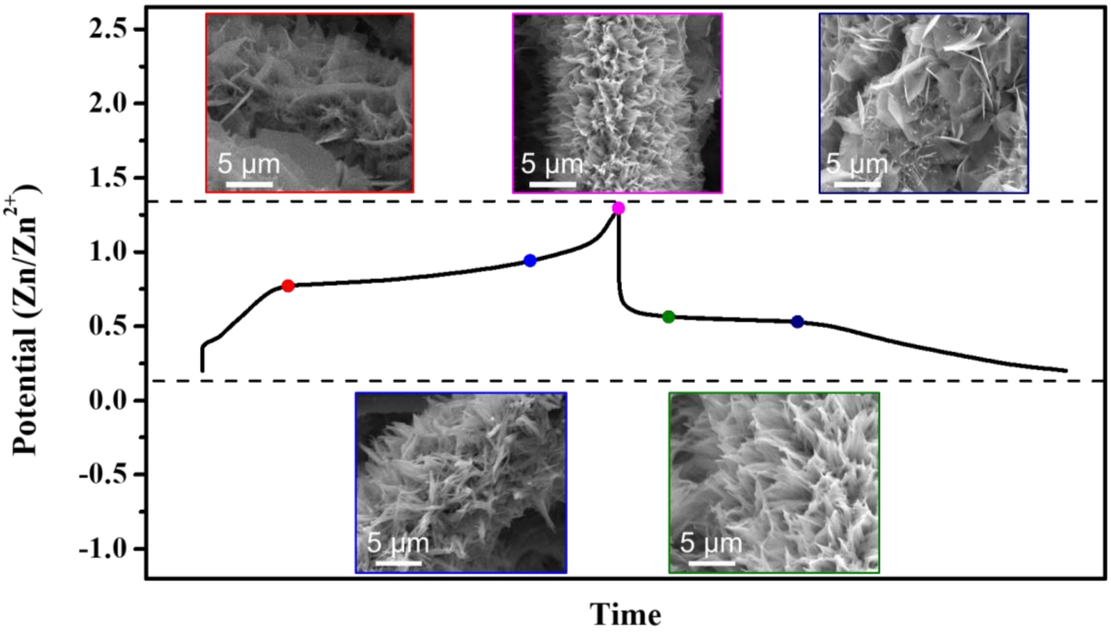

Figure S8. Ex-situ SEM images of cathode at different voltages in one charge/discharge cycle.

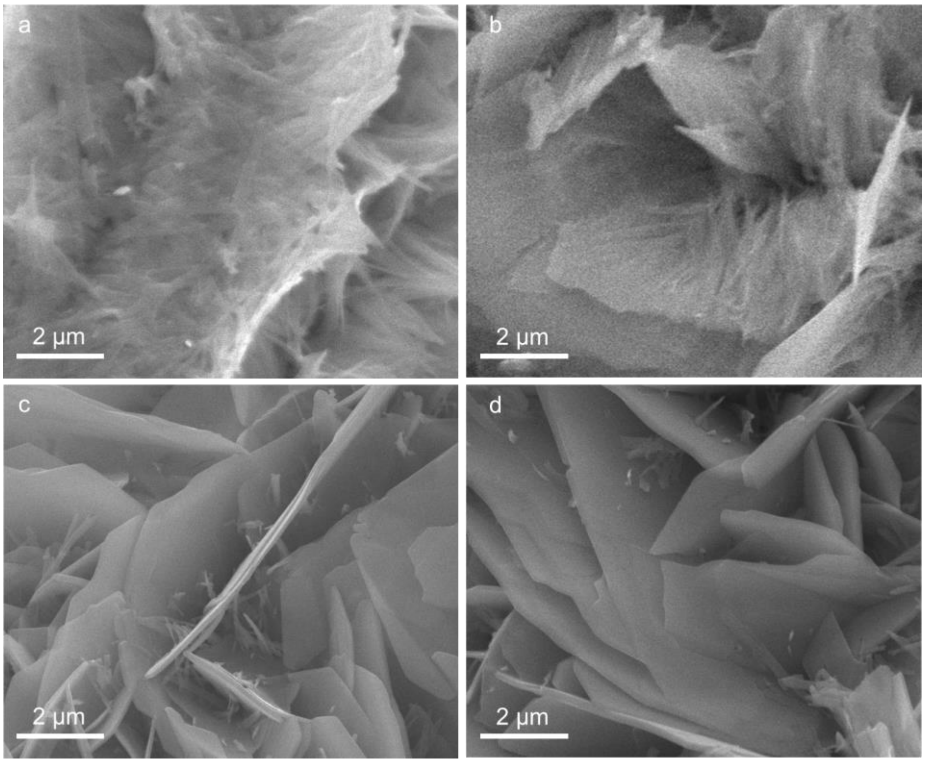

Figure S9. SEM of insertion state cathode discharge at (a) 4 A g^{-1}, (b) 1.6 A g^{-1}, (c) 0.8 A g^{-1} and (d) 0.4 A g^{-1}.

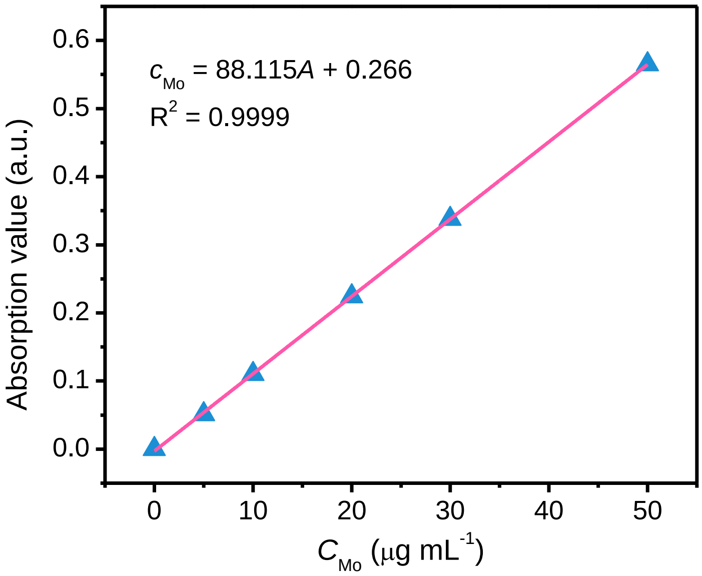

Figure S10. Standard curve of UV-Visible spectra for quantitative analyse of concentration of Mo species (C_{Mo}).

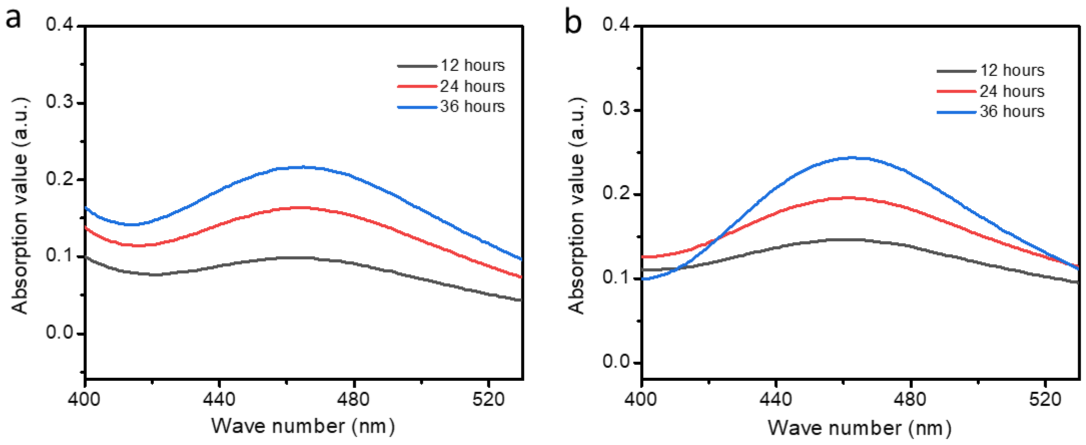

Figure S11. UV-Visible spectra of aqueous electrolyte after (a) pristine and (b) extraction-state MoO_3 were immersed for different time.

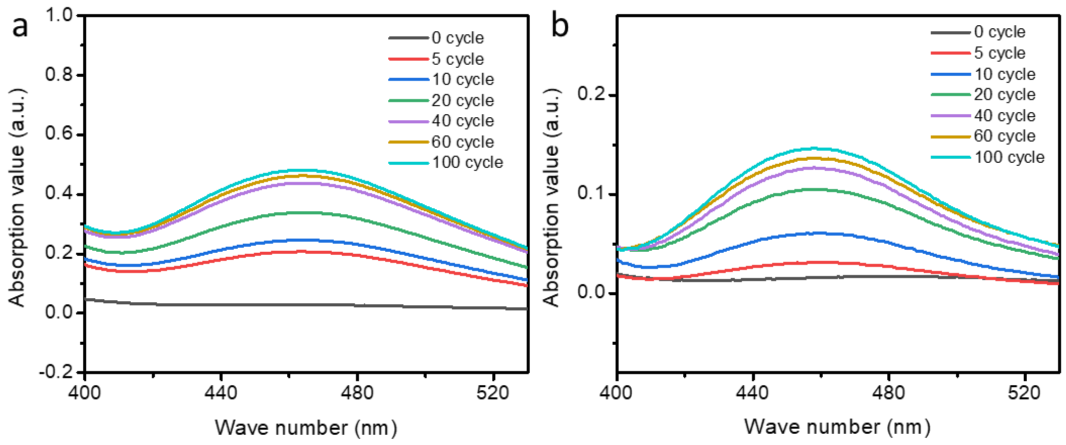

Figure S12. UV-Visible spectra of (a) aqueous electrolyte and (b) quasi-solid-state electrolyte after different charge-discharge cycles.

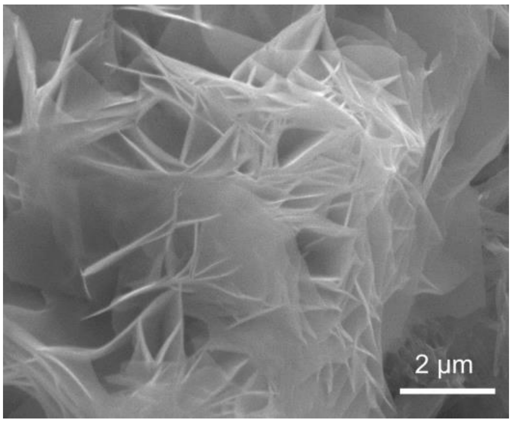

Figure S13. SEM image of insertion-state MoO_3 in quasi-solid-state electrolyte.

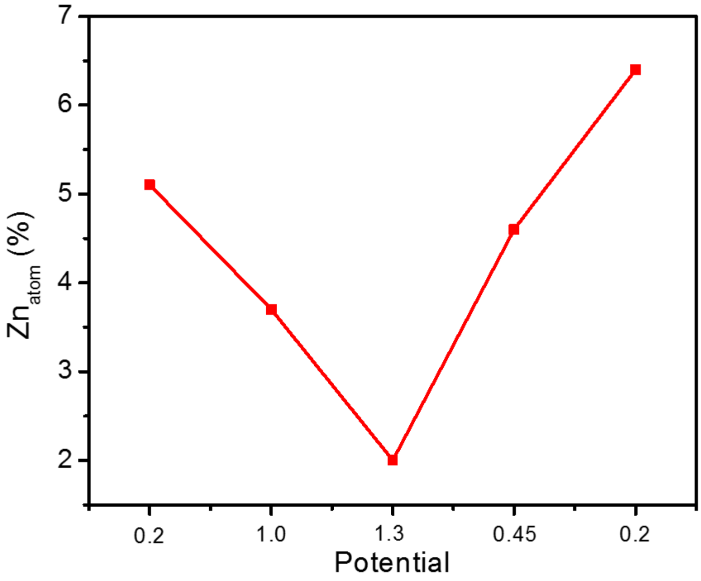

Figure S14. Zinc atom content of cathode in quasi-solid-state electrolyte at different voltages in first charge/discharge cycle.

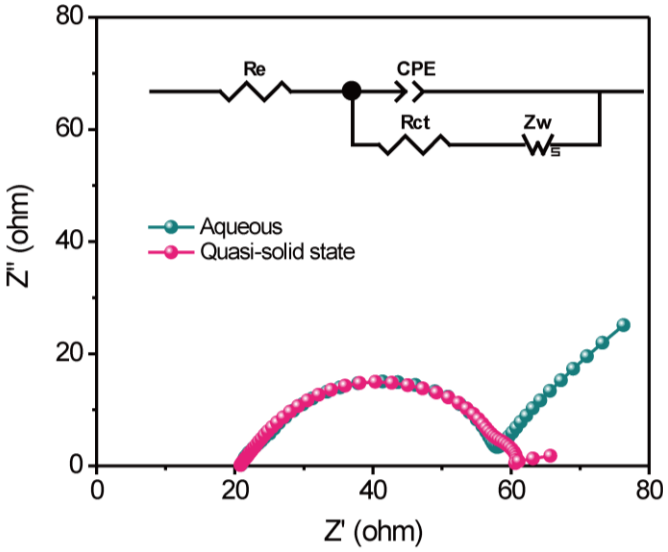

Figure S15. Nyquist plots of the aqueous and quasi-solid-state Zn//MoO_3 batteries. The inset shows the equivalent-circuit diagram.

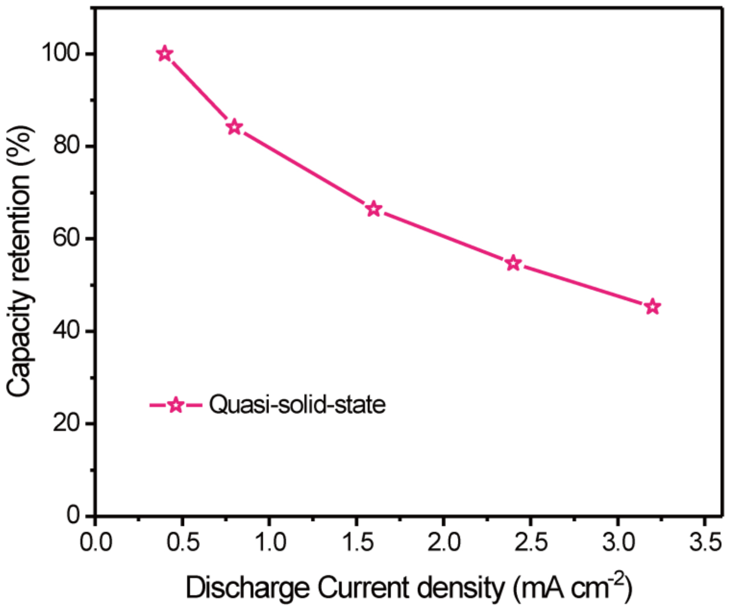

Figure S16. Rate performance of the quasi-solid-state device.

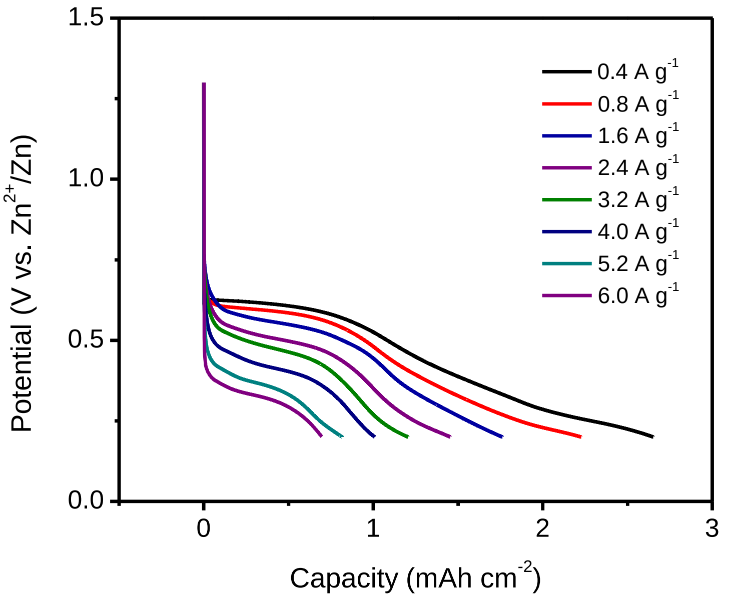

Figure S17. Capacity of the quasi-solid-state battery under different current densities (1C = 0.4 A g^{-1}).
